# The Associations between COMT and MAO-B Genetic Variants with Negative Symptoms in Patients with Schizophrenia

**DOI:** 10.3390/cimb43020045

**Published:** 2021-07-08

**Authors:** Zoran Madzarac, Lucija Tudor, Marina Sagud, Gordana Nedic Erjavec, Alma Mihaljevic Peles, Nela Pivac

**Affiliations:** 1 Department of Psychiatry and Psychological Medicine, University Hospital Centre Zagreb, 10 000 Zagreb, Croatia; zoranmadzarac@gmail.com (Z.M.); marinasagud@mail.com (M.S.); apeles@mef.hr (A.M.P.); 2 Ruder Boskovic Institute, 10 000 Zagreb, Croatia; lucija.tudor@irb.hr (L.T.); gnedic@irb.hr (G.N.E.); 3 School of Medicine, University of Zagreb, 10 000 Zagreb, Croatia

**Keywords:** alogia, anhedonia, COMT, MAO-B, negative symptoms, patients, polymorphisms, schizophrenia, sex-differences

## Abstract

Negative symptoms of schizophrenia, including anhedonia, represent a heavy burden on patients and their relatives. These symptoms are associated with cortical hypodopamynergia and impaired striatal dopamine release in response to reward stimuli. Catechol-O-methyltransferase (COMT) and monoamine oxidase type B (MAO-B) degrade dopamine and affect its neurotransmission. The study determined the association between *COMT* rs4680 and rs4818, *MAO-B* rs1799836 and rs6651806 polymorphisms, the severity of negative symptoms, and physical and social anhedonia in schizophrenia. Sex-dependent associations were detected in a research sample of 302 patients with schizophrenia. In female patients with schizophrenia, the presence of the G allele or GG genotype of *COMT* rs4680 and rs4818, as well as GG haplotype rs4818-rs4680, which were all related to higher COMT activity, was associated with an increase in several dimensions of negative symptoms and anhedonia. In male patients with schizophrenia, carriers of the *MAO-B* rs1799836 A allele, presumably associated with higher MAO-B activity, had a higher severity of alogia, while carriers of the A allele of the *MAO-B* rs6651806 had a higher severity of negative symptoms. These findings suggest that higher dopamine degradation, associated with COMT and MAO-B genetic variants, is associated with a sex-specific increase in the severity of negative symptoms in schizophrenia patients.

## 1. Introduction

Schizophrenia is often a chronic, disabling mental disorder characterized by a great heterogeneity in clinical symptoms, treatment response, course of the disease and impact on patient’s functioning [1]. It has been analyzed through psychopathological dimensions, including positive (delusions, hallucinations), disorganization (thought disorder, inappropriate affect, disorganized behavior) and negative dimensions [2]. The negative symptoms of schizophrenia refer to the diminishing or absence of normal behaviors related to motivation and interest (avolition, anhedonia, asociality) or expression (blunted affect, alogia) [3]. Due to their different etiologies, they are divided into primary and secondary negative symptoms. Primary negative symptoms are among the core disease symptoms, characterized by an early onset which typically develops prior to treatment commencement.

In contrast, secondary negative symptoms have other causes. They may originate from positive symptoms (such as social withdrawal due to persecutory delusions), antipsychotic treatment (i.e., sedation, bradykinesia or amotivation), social deprivation (particularly in institutionalized patients), and other associated depressive symptoms. [4]. Prevalence of negative symptoms is high, with up to 60% of schizophrenia patients developing clinically relevant negative symptoms that require treatment [5]. They can occur at any point during the course of the illness. In up to 90% of patients with first-episode psychosis, at least one negative symptom can be observed. Furthermore, in 35–70% of patients, clinically significant negative symptoms persist after treatment [6]. In contrast to positive and secondary negative symptoms, primary negative symptoms are lacking in effective treatment options, with most currently available drugs having little to no effect on them [7,8]. According to the dopaminergic hypothesis of schizophrenia, negative symptoms, together with the loss of motivation and impairment in cognition, are consequences of diminished dopaminergic function in the frontal lobe and additional mesolimbic structures [9].

Anhedonia refers to a reduced ability or capacity to feel pleasure from otherwise pleasurable experiences [10]. Anhedonia may develop across different mental disorders but is more commonly observed in individuals with major depression and schizophrenia than in healthy individuals [11]. The two sub-types are physical anhedonia, an inability to experience physical pleasure, and social anhedonia, a reduced capacity to feel pleasure in social situations [12]. While in patients with depression, anhedonia represents a state-marker [13], it also corresponds to negative symptoms in schizophrenia and is considered to be a trait-marker of the disease due to its unremitting nature [13,14,15]. Anhedonia is assumed to be related to deficits in mesolimbic dopamine transmission [16], and higher subcortical dopamine levels are associated with a lower intensity of negative symptoms, including anhedonia, in patients with schizophrenia [17]. As dopamine activity is terminated by the two degrading enzymes, catechol-O-methyltransferase (COMT) and monoamine oxidase (MAO), the presence and intensity of anhedonia may be associated with the rate of dopamine metabolism, which, in part, is dependent on the polymorphisms inside *COMT* and *MAO* genes. However, those two enzymes contribute to dopamine metabolism in cortical and subcortical regions differently. Therefore, the two dopamine-degrading enzymes, COMT and MAO, play a significant role in maintaining normal dopaminergic function.

Catechol-O-methyltransferase particularly modulates dopaminergic transmission in the prefrontal cortex (PFC), with pharmacological manipulation studies showing that it plays an important role in PFC-mediated cognition [18]. *COMT* genes contain numerous polymorphisms, and the most frequently studied of them is a functional single nucleotide polymorphism (SNP) rs4680 (G/A or Val/Met substitution) that affects enzymatic activity [19] and the rs4818 (C/G or Leu/Leu substitution) polymorphism that affects COMT expression [20]. The *COMT* rs4680 polymorphism impairs the thermostability of the mature protein, causing alterations in dopamine levels in different brain regions, specifically in the PFC, with the G allele being a predominant factor of higher COMT activity in the PFC, leading to lower synaptic dopamine levels and a disturbance in prefrontal function [21]. While the G allele was reported to be associated with schizophrenia [22], a lack of any association has also been reported [23]. Different alterations in cognition and neuronal functions among patients with schizophrenia have been related to the presence of the G allele [24]. Moreover, an association between the rs4680 polymorphism and structural brain volumes in healthy subjects and patients with schizophrenia was reported [25,26], but also denied [27]. A possible reason for these conflicting results could be a sexually divergent effect of the *COMT* polymorphism on subcortical structures in schizophrenia. This theory is supported by evidence of smaller volumes of the right caudate and bilaterally smaller volumes in the putamen, pallidum, and nucleus accumbens found in female schizophrenia patients who were carriers of the AA rs4680 genotype [28]. Considering the above-mentioned association of rs4680 with structural brain abnormalities which have been found to affect the development of negative symptoms of schizophrenia [29], it is not surprising that rs4680 also showed a significant association, with a blunted effect [30].

The rs4818 polymorphism was suggested to be responsible for an even larger variation in the COMT activity than the rs4680 polymorphism [31]. The G allele of the *COMT* rs4818 polymorphism, similar to rs4680, is also associated with higher COMT activity and, therefore, lower prefrontal dopaminergic function [20]. The rs4818 polymorphism is often transmitted with *COMT* rs4680 polymorphisms in a haploblock [32,33], and studies dealing with the association of *COMT* haplotypes and schizophrenia, or its symptoms, often report contradictory findings [34,35]. Nonetheless, a haplotype of the *COMT* rs4818 and rs740603 polymorphisms was reported to be linked to negative symptoms of schizophrenia [35].

Monoamine oxidase is a mitochondrial enzyme existing in two biochemically distinct forms, MAO-A and MAO-B, which are encoded by distinct genes, located adjacently on the X chromosome in opposite directions [36]. Despite being expressed predominantly in glial cells, it has an important role in regulating the activity of dopaminergic neurons. Dopaminergic neurons in the substantia nigra are surrounded by astrocytes containing high levels of MAO-B [37]. In the mice model of Parkinson’s disease, MAO-B over-expression in astrocytes exacerbated Parkinsonian motor dysfunction, in comparison to wild-type mice [38]. In addition, MAO-B, while less abundant than MAO-A, is also present in dopaminergic neurons of the substantia nigra, where cytosolic dopamine competes between vesicular monoamine transporters and MAO-B [39]. In accordance with the dopaminergic theory of schizophrenia, low activity of MAO could be a risk factor in the development of the disorder itself [40]. Besides its role in schizophrenia in general, MAO-B has an important role in the development of the negative symptoms of schizophrenia, which is evident from a study reporting the positive effects of selegiline, a selective MAO-B inhibitor, on the treatment of negative symptoms in schizophrenia [41]. The rs1799836 (A to G substitution) polymorphism of the *MAO-B* gene was found in all human populations, and it was suggested that this affects MAO-B expression with the A allele, increasing the efficiency of the splicing process, leading to an increased MAO-B protein expression [42]. The *MAO-B* rs1799836 polymorphism was reported to be associated with the etiology of schizophrenia [43], and it was also associated with affective flattening, but only in female schizophrenia patients [44].

The other polymorphism, an *MAO-B* rs6651806 (A to C substitution), was studied in healthy human volunteers and its association with personality traits relating to positive and negative affect was investigated, with the results indicating a significant association between negative emotionality and rs6651806 [45]. In addition, the same polymorphism was found to be associated with higher or lower levels of 3-methoxy-4-hydroxyphenylglycol, one of the major monoamine metabolites in the central nervous system, measured in the cerebrospinal fluid of psychotic men [46].

Although the aforementioned studies demonstrated the link between *COMT* rs4680 and rs4818 and *MAO-B* rs1799836 polymorphisms in schizophrenia and, more specifically, with negative symptoms, their association with anhedonia has been addressed in only two studies to date. Namely, *COMT* rs4680 polymorphisms have been studied regarding anhedonia only in healthy individuals, where it was associated with reward learning [47]. In addition, *MAO-B* rs1799836 was investigated in Mexican patients with schizophrenia in relation to negative symptoms, where anhedonia was measured as a single item [44]. Anhedonia is, however, a complex symptom, which is composed of both physical and social components. There are no data on the relationship between *COMT* rs4818 and anhedonia in any population, whereas the *MAO-B* rs6651806 polymorphism, to the best of our knowledge, has not been investigated in schizophrenia. In view of the importance of dopaminergic neurotransmission in hedonic behavior and the role of dopamine-metabolizing enzymes in maintaining the optimal dopaminergic brain functioning, the aim of the present study was to investigate the association between *COMT* rs4680 and rs4818 and *MAO-B* rs1799836 and rs6651806 polymorphisms and the presence and severity of physical and social anhedonia in patients with schizophrenia.

## 2. Materials and Methods

### 2.1. Participants

The study sample consisted of 302 patients who met the DSM-IV criteria [48] for schizophrenia, who were treated in the Department for Psychiatry and Psychological Medicine at the University Hospital Centre Zagreb. Included were (1) participants with schizophrenia, (2) aged between 18 and 65 years, (3) who signed an informed consent document approved by the local Ethics Committee. Excluded were patients with (1) any neurological condition which may influence hedonic capacity, such as Parkinson’s disease, (2) prior head trauma, (3) cognitive difficulties that compromised the capacity to read, understand, or fill out self-rating questionnaires, (4) current severe somatic disease, (5) recent (less than 3 months ago) alcohol or psychoactive drug abuse or dependence (except caffeine and nicotine use) and/or (6) severe psychotic, aggressive or psychomotor symptoms, which could preclude cooperation during testing.

### 2.2. Procedures

Diagnosis of schizophrenia was confirmed by a structured clinical interview [49] based on the DSM-IV criteria [48]. Symptoms of schizophrenia were assessed by the Positive and Negative Syndrome Scale (PANSS) [50]. This widely used instrument has 30 items, divided into positive (7 items), negative (7 items), and general psychopathology (16 items) subscales. Each item describes the symptom severity from 0 (absent) to 7 (most severe). Negative symptoms were appraised by the Clinical Assessment Interview for Negative Symptoms (CAINS) [51] and the Brief Negative Symptom Scale (BNSS) [52]. Given the limitations of PANSS in measuring negative symptoms, these two physician-rated scales were constructed to match the current concept of negative symptoms, according to the 2005 expert consensus [53]. The CAINS is composed of a 19-item motivation and pleasure scale, which measures motivation and interest for (1) social, (2) vocational, and (3) recreational activities, and a four-item expression scale, which records the deficits in expression. Each item is rated on a scale from 0 to 4, ranging from absent to severe. The BNSS consists of 13 items, which are measured on a from 0 to 7 scale, with the higher scores denoting greater deficits. Its five subscales evaluate five negative symptom domains: anhedonia, asociality, avolition, alogia, and blunted affect while the 6th subscale estimates the lack of normal distress [52].

The severity of depressive symptoms was tested by the Calgary Depression Scale for Schizophrenia (CDSS) [54]. This is a 9-item scale, with each item graded from 0 (absent) to 3 (severe). A total score higher than 6 indicates the presence of a major depressive episode. General functioning was scored by the Global Assessment of Functioning (GAF) [55]. This measures the impact of symptoms on functioning in social, occupational and psychological areas, on a continuous scale from 1, referring to the most severe dysfunction, to 100, representing superior functioning. Anhedonia was assessed with two self-rating instruments: the Revised Physical Anhedonia Scale (RPAS) and the Revised Social Anhedonia Scale (RSAS), [12]. The RPAS assesses for deficits in experiencing pleasure from food, sex, and other activities or scenarios. It consists of 61 items represented in a “true” or “false” format. The RSAS scale measures hedonic deficits in non-physical stimuli, such as engaging in social contact. It includes 40 items, also rated as “true” or “false”. Depending on the answers from the two anhedonia scales, respondents were considered to have PA if they had RPAS scores > 20 in women and >28 in men, while the cut-off scores for SA were >16 for women and >20 for men [56].

### 2.3. Blood Sampling and Genotyping

Blood samples (approximately 8 mL) were collected into BD Vacutainer™ glass blood collection tubes (Becton, Dickinson and Company, Franklin Lakes, NJ, USA) with acid citrate dextrose (ACD), after overnight fasting. Genomic DNA was isolated from blood samples using a salting out method [57], while genotyping was performed using Applied Biosystems^®^ 7300 Real-Time PCR System with TaqMan^®^ SNP Genotyping Assay primers and probes: C___2538750_10 (rs4818), C__25746809_50 (rs4680), C___8878790_10 (rs1799836) and C__29047318_10 (rs6651806) (Applied Biosystems, Foster City, CA, USA) following manufacture’s protocol.

### 2.4. Statistical Analysis

Statistical analysis was performed in Graph Prism version 7.00 (GraphPad Software, Inc., La Jolla, CA, USA). The normality of distribution was tested with the Kolmogorov–Smirnov test. Due to deviation from normal distribution for clinical scales, nonparametric statistical tests were used in the analysis. The data are represented with median and interquartile range (25th and 75th percentile), or with boxplot in graphic representation. The Mann–Whitney test and Kruskal–Wallis ANOVA were used to analyze differences in psychometric and clinical scores between two or more than two groups, respectively. A Chi-square test was used to test the difference in the prevalence of genotypes and haplotypes between two or more groups of subjects [58].

Haplotype analysis for *COMT* rs4818 and rs4680 and *MAO-B* rs1799836 and rs6651806 was performed using Haploview v. 4.2 [59] and PLINK v. 1.07 software [60]. SNPs were considered to be in linkage disequilibrium if D’ was >0.80. Tested polymorphisms of both genes had D’ = 99 and D’ = 96, respectively, so the most probable haplotype pair for each patient was determined using expectation maximization algorithm (EM algorithm). Since both of the MAO-B polymorphisms are located on the X chromosome, genetic and allelic analyses regarding MAO-B polymorphisms were performed in male and female patients separately.

G* Power 3 Software [61] was used to determine a priori sample size and statistical power (with α = 0.05; with expected small to medium effect size = 0.2; power (1 − β) = 0.800). The required sample sizes were for a χ2-test *n* = 273 (for df = 3) and *n* = 241 (for df = 2); for Kruskal–Wallis ANOVA, the required sample size was *n* = 246 for 3 groups; for Mann–Whitney test, the required sample size was *n* = 260. As the study sample included *n* = 302 patients, the study had an adequate sample size and statistical power. All tests were two-tailed.

## 3. Results

### 3.1. Demographic and Clinical Data

The study included a total of 302 Caucasian patients with schizophrenia with an average age of 42 (35;51) years and average PANSS total scores of 68 (59;79). Table 1 represents demographic and clinical data for 178 male (58.9%) and 124 female (41.1%) patients, with schizophrenia included in the study. Female patients were slightly older than the male patients (*p* = 0.016), while male subjects were more likely to be smokers (*p* = 0.006), regular alcohol consumers (*p* = 0.027) and former recreational drug users (*p* < 0.001) than female patients (Table 1). On the other hand, there were no differences in the dosage of antipsychotic therapy specified in chlorpromazine equivalents (*p* = 0.771), nor in the type of antipsychotic therapy used in treatment (*p* = 0.328). The percent of patients who had a history of suicide attempt(s) was similar between sexes (*p* = 0.762). Approximately 21.9% of male subjects tried to commit suicide at least once in a lifetime, compared to 23.4% of female patients (Table 1).

The severity of positive and negative symptoms of schizophrenia (PANSS total scores) (*p* = 0.653), negative symptoms evaluated with the Clinical Assessment Interview for Negative Symptoms (CAINS) (*p* = 0.129), the Brief Negative Symptom Scale (BNSS) (*p* = 0.141), depressive symptoms estimated with the Calgary Depression Scale for Schizophrenia (CDSS) (*p* = 0.080), as well as global functionality assessed with the Global Assessment of Functioning Scale (GAF) (*p* = 0.979), were similar between sexes (Table 1). However, male patients had significantly higher total scores on the Revised Physical Anhedonia Scale (RPAS) than female patients (*p* = 0.02. Revised Social Anhedonia Scale (RSAS) scores (*p* = 0.571) did not differ between sexes. When patients were divided depending on the presence or absence of physical (minimum of 28 point on RSAS for men and 20 points for women) and social anhedonia (minimum of 20 point on RSAS for men and 16 points for women), women met the criteria for physical (*p* = 0.003) and social (*p* = 0.007) anhedonia more often than men (Table 1).

A general linear model was used to determine the effect of demographic and clinical data (patient age, sex, smoking, alcohol consumption, history of drug abuse, history of suicide attempts and therapeutic dose of antipsychotic in chlorpromazine equivalents) on the CAINS, BNSS, RPAS and RSAS total scores (Table 2). There was a significant positive correlation between age and severity of physical (*p* = 0.048) and social anhedonia (*p* = 0.035) and significant effect of sex (*p* = 0.012) on physical anhedonia. Smoking was significantly associated with RSAS scores, whereas non-smokers had more prominent symptoms of social anhedonia than smokers (*p* = 0.039). The CAINS (*p* < 0.001) and BNSS scores (*p* = 0.001), which reflect the severity of negative symptoms in schizophrenia, were significantly associated only with antipsychotic dose, which was the expected outcome. Alcohol consumption and history of suicide attempts did not affect scores on any of the tested scales (Table 2).

### 3.2. Association of COMT Polymorphisms with Negative Symptoms in Schizophrenia

The minor (alternative) allele frequency for *COMT* rs4680 polymorphism (A allele) in patients with schizophrenia was 0.498 and 0.399 for *COMT* rs4818 polymorphism (C allele) which is consistent with estimated MAF in European populations (alternative allele frequency (A allele) = 0.508 for rs4680 and MAF (C allele) = 0.391 for rs4818) (ALFA project) (Supplementary Table S1). The distribution of genotypes of both polymorphisms was similar between male and female subjects (χ^2^ = 4.421; df = 1; *p* = 0.110 for rs4680 and χ^2^ = 4.857; df = 2; *p* = 0.088 for rs4818), as well as rs4818 alleles (χ^2^ = 3.481; df = 2; *p* = 0.062). However, the G allele of rs4680 was significantly more prevalent in female patients (54.8%) and less common in male subjects (46.3%) than the A allele (χ^2^ = 4.215; df = 1; *p* = 0.040) (Supplementary Table S1).

Haplotype analysis showed high linkage disequilibrium between the two *COMT* polymorphisms, rs4818 and rs4680 (D’ = 99; LOD > 2); thus, the haplotype pair for each patient was estimated and included in the subsequent analysis. The most common haplotype was CA, with 48.6% prevalence in the total sample, followed by GG with 38.6% and GA with 9.8%. The rarest haplotype (GA) was prevalent in less than 1% of the total sample (0.2%) and was excluded from the analysis. The distribution of COMT haplotypes (χ^2^ = 4.289; df = 2; *p* = 0.117) was not different between male and female patients (Supplementary Table S1).

In female patients, both *COMT* rs4680 and rs4818 were associated with negative symptoms, evaluated with the CAINS scale, whereas the G allele of both SNPs was associated with higher scores compared to the A allele of rs4680 (*p* = 0.015) and the C allele of rs4818 (*p* = 0.006) (Table 3). Additionally, the G allele of rs4680 and rs4818 was associated with higher scores on the CAINS subscales for socialization (U = 6144.0; *p* = 0.008 for rs4680, U = 5931.0; *p* = 0.003 for rs4818), work (U = 6499.0; *p* = 0.044 for rs4680, U = 6076.0; *p* = 0.006 for rs4818) and recreation (U = 6384.0; *p* = 0.025 for rs4680; U = 6349.0; *p* = 0.024 for rs4818) (Supplementary Table S2). Other scales (RPAS and RSAS), including the BNSS total scores, were not associated with any of the polymorphisms. A higher BNSS anhedonia subscale score was associated with both the rs4680 G allele (U = 6336.0; *p* = 0.022, allelic model) and rs4818 G allele (U = 6119.0; *p* = 0.008). Higher BNSS total scores in female patients were associated with the GG haplotype (H = 7.0421; df = 2; *p* = 0.030) (Table 3, Supplementary Table S3).

The χ^2^ test was used to determine the differences in the prevalence of rs4680 and rs4818 genotypes and alleles, and their haplotypes, depending on the presence of physical and social anhedonia in male and female patients with schizophrenia (Supplementary Table S4). Neither of the polymorphisms, nor their haplotype, were differentially distributed between male patients with or without physical and social anhedonia. In female patients, rs4680 was not associated with anhedonia prevalence but significant changes in distribution were seen in the frequency of the rs4818 G allele, which was more prevalent in female subjects with social anhedonia (54.2%) when compared to patients without it, where the frequency of the G allele was 40.3% (χ^2^ = 3.957; df = 1; *p* = 0.047) (Supplementary Table S4). Haplotype analysis did not show an association between the *COMT* rs4818-rs4680 haplotype and the presence of physical or social anhedonia in male or female patients (Supplementary Table S4).

### 3.3. Association of MAO-B Polymorphisms with Negative Symptoms in Schizophrenia

The minor (alternative) allele frequency for *MAO-B* rs1799836 polymorphism (G allele) in patients with schizophrenia was 0.483, and for *MAO-B* rs6651806 (C allele) 0.300, similar to the European population were MAF (G allele) = 0.455 and MAF (C allele) = 0.291 for rs1799836 and rs6651806 SNP, respectively) (ALFA project) (Supplementary Table S1). No differences in the prevalence of alleles were seen between sexes (χ^2^ = 0.030; df = 1; *p* = 0.860 for rs1799836 and χ^2^ = 3.481; df = 1; *p* = 0.062 for rs6651806).

*MAO-B* polymorphisms rs1799836 and rs6651806 were in high linkage disequilibrium (D’ = 96; LOD > 2) and, therefore, a haplotype pair was estimated for each subject. The most common haplotype was AA, with 51.2% prevalence in the total sample, followed by GC with 29.9% and GA with 18.5%, while the rarest haplotype (AC) was found in less than 1% of the total sample (0.5%) and was excluded from the subsequent analysis. The distribution of *MAO-B* haplotypes (χ^2^ = 1.947; df = 2; *p* = 0.378) did not differ between male and female patients (Supplementary Table S1).

Total scores on the RPAS, RSAS, CAINS and BNSS scales were not associated with rs1799836 polymorphism in male or in female patients with schizophrenia (both codominant and allelic model) (Table 4). However, male patients carrying the A allele of *MAO-B* rs1799836 had significantly higher scores on the BNSS alogia subscale than male patients carrying the G allele (U = 2765.0; *p* = 0.026). This result was not confirmed in female subjects (H = 3.266; df = 2; *p* = 0.195, codominant model; U = 7180.5; *p* = 0.989, allelic model) (Supplementary Table S5). *MAO-B* rs6651806 was significantly associated with the CAINS (U = 2212.5; *p* = 0.036) and the BNSS (U = 2105.0; *p* = 0.013) total scores in male patients, but not in female patients (Table 4). Additionally, the CAINS recreation (U = 2223.0; *p* = 0.035) and expression (U = 2132.0; *p* = 0.017) subscales and BNSS anhedonia (U = 2208.5; *p* = 0.034) and alogia (U = 1949.5; *p* = 0.002) subscales were associated with *MAO-B* rs6651806 in male patients. In all cases, the A allele was associated with the higher scores, i.e., the higher severity of negative symptoms evaluated with the CAINS or BNSS in male patients compared to the C allele carriers (Supplementary Tables S5 and S6). These results were not observed in female patients. However, female A allele carriers of rs6651806 had significantly higher symptoms of social anhedonia compared to the C allele carriers (U = 5115.5; *p* = 0.031) (Table 4). Haplotype analysis revealed a significant association between the GC haplotype and lower scores on the CAINS expression subscale (H = 6.308; df = 2; *p* = 0.043), BNSS scale (H = 7.229; df = 2; *p* = 0.027) and BNSS alogia subscale (H = 9.047; df = 2; *p* = 0.011) compared to the AA and GA haplotype carriers, but only in male subjects (Table 4, Supplementary Tables S5 and S6).

There was no difference in the distribution of rs1799836 genotypes or alleles between male (χ^2^ = 0.121; df = 1; *p* = 0.728 for physical and χ^2^ = 0.384; df = 1; *p* = 0.535 for social anhedonia) or female subjects (χ^2^ = 2.672; df = 1; *p* = 0.102 for physical and χ^2^ = 2.405; df = 1; *p* = 0.121 for social anhedonia), depending on the presence of physical and social anhedonia (Supplementary Table S7). Prevalence of rs6651806 A allele was higher in female patients with physical (χ^2^ = 4.201; df = 2; *p* = 0.040) and social anhedonia (χ^2^ = 3.902; df = 1; *p* = 0.048) compared to female patients who did not meet the criteria for physical and social anhedonia (less than 20 points on RPAS and 16 on RSAS) (Supplementary Table S7), but these results were not replicated in male patients (χ^2^ = 1.786; df = 1; *p* = 0.181 for physical, and χ^2^ = 0.065; df = 1; *p* = 0.798 for social anhedonia). Haplotype analysis did not show a significant association between MAO-B rs1799836-rs6651806 haplotypes and the prevalence of physical and social anhedonia in patients with schizophrenia unrelated to sex (Supplementary Table S7).

## 4. Discussion

The main findings of this study are: (1) Female patients with schizophrenia with *COMT* rs4680 GG genotype had higher CAINS socialization subscale scores, while G allele carriers had higher scores for CAINS socialization, vocational and recreational subscale scores, as well as BNSS anhedonia subscale scores; (2) Female patients with *COMT* rs4818 GG genotype demonstrated a greater CAINS total, socialization and vocational scores, and BNSS anhedonia ratings, similarly to G allele carriers, compared to C carriers, who additionally had greater CAINS recreational scores; (3) Female patients who are carriers of the rs4818-rs4680 GG haplotype had a higher CAINS total score and CAINS socialization and vocational subscale scores, as well as greater BNSS anhedonia level, compared to CA and CG haplotype carriers; (4) *MAO-B* rs1799836 A carriers were associated with higher BNSS alogia subscale scores compared to G carriers in male patients; (5) Female patients, carriers of the A allele of the *MAO-B* rs6651806, had social anhedonia and physical anhedonia more frequently and greater RSAS scores than carriers of the C allele; (6) Male patients with schizophrenia who were *MAO-B* rs6651806 A allele carriers had higher CAINS and BNSS total scores, greater CAINS recreational and expression scores, and greater BNSS anhedonia and alogia subscale scores than C allele carriers; (7) Male patients who are carriers of the rs1799836-rs6651806 GC haplotype had lower CAINS expression, BNSS total scores, and alogia subscale scores.

The study was carried out in a sample with clinically stable conditions, as reflected by the patient median PANSS total scores of 68. About one third of the participants were employed. The results highlight sex-related differences in all significant associations between dopamine-degrading enzyme polymorphisms, negative symptoms of schizophrenia, and social and physical anhedonia. Such distinct findings may be explained by different demographic and clinical features, sexual dimorphism of the COMT and MAO-B enzymes, or both. However, those discrepancies cannot be attributed to differences in the present disease features, since the severity of overall negative and depressive symptoms, functionality, suicide attempt history, and antipsychotic doses were similar across sexes. Regardless, female patients with schizophrenia had almost twice as many incidences of physical and social anhedonia, while the intensity of physical anhedonia was milder when compared to male patients. It may be presumed that physical anhedonia, though more common in women, is more severe in men, and this occurred despite the fact that female patients were older, and the age positively correlated with anhedonia severity. There is meta-analytic evidence of the greater severity of both physical anhedonia and social anhedonia among men, at least in healthy populations [62], and of higher intensity of social anhedonia in men from a large, population-based international sample [63]. On the contrary, the intensity of social anhedonia in our trial was similar across sexes. Given that our male patients were more frequently smokers, and that smoking was correlated with the severity of social anhedonia but not physical anhedonia in a general linear model, tobacco use may have reduced social anhedonia in male patients to levels similar to those seen in female patients with schizophrenia. However, data on smoking were not provided in the aforementioned studies [62,63].

Our findings suggest the association between *COMT* rs4680 G variant, which is related to greater COMT activity [21] and greater protein abundance and stability [19], with decreased motivation and interest in social and vocational activities and BNSS-measured anhedonia in female patients. While there are no data on the relationship between this polymorphism and physical anhedonia and social anhedonia in patients with schizophrenia, our results disagree with the highest levels of physical anhedonia observed in the carriers of the GG homozygous genotype among the first-degree relatives of patients with schizophrenia [64]. The discrepancies may arise from the small sample size, lower severity of anhedonia, and history of depressive disorders in some participants [64]. Nevertheless, the G carriers had a higher severity of BNSS-measured anhedonia.

Strikingly, very similar findings [20] to ours were observed for the *COMT* rs4818 polymorphism, given that either homozygous individuals or carriers for COMT high-activity G variant had worse CAINS total, socialization and vocational scores, and BNSS anhedonia ratings. However, this effect was detected only in a group of female patients with schizophrenia. In addition, female patients with schizophrenia who were G allele carriers had higher prevalence of social anhedonia. Dopamine is predominantly involved in the anticipatory part of hedonic experience [65]; patients with schizophrenia typically experience deficits in the anticipatory part of anhedonia while the consummatory component remains preserved [66]. Additionally, BNSS refers to both anticipatory and consummatory constituents of anhedonia [52]; RPAS focuses mostly on the consummatory anhedonia [67], while RSAS measures the anticipatory anhedonia along with consummatory anhedonia [68]. Taking the connection between social anhedonia and reduced self-efficiency into consideration, the evidence shows that anticipatory and consummatory anhedonia relate to different brain systems. For example, while consummatory anhedonia was associated with decreased ventral striatal activity, anticipatory anhedonia was related to fronto-striatal pathway deficits (except from the ventral striatum) [69]. Of note, COMT is a dominant dopamine-breaking enzyme in the prefrontal cortex [70]; therefore, its greater efficacy is associated with lower prefrontal synaptic dopamine levels [21,71]. On the other hand, the COMT role in the striatum appears less prominent due to an abundance of dopaminergic transporters which clear up synaptic dopamine levels. Preclinical studies have shown that genetically determined high COMT activity in the frontal cortex is related to a greater striatal dopamine release [72], whereas low COMT activity, while rising frontal dopamine levels, do not change striatal dopamine concentration [73]. Based on this data, it may be speculated that genetically-induced higher COMT activity may be related to higher levels of anticipatory anhedonia, measured by the BNSS and RSAS, but not physical anhedonia, assessed by the RPAS, in female patients with schizophrenia. This must be interpreted with great caution, given that neither anticipatory nor consummatory anhedonia were specifically addressed in this study, nor was dopamine activity measured in different brain regions. In agreement, female patients with schizophrenia with rs4818-rs4680 GG haplotype, which is associated with the greatest COMT activity, compared to other haplotype carriers, had higher CAINS total scores, socialization and vocational subscale scores, and greater BNSS anhedonia levels, which underscores the association between prefrontal COMT activity and specific negative symptoms.

All of our findings were observed exclusively in female patients with schizophrenia. Sexual dimorphism was previously reported in the COMT activity in cerebellum [74] and dorsolateral prefrontal cortex [21]. Moreover, *COMT* rs4680 polymorphism was differently correlated with subcortical brain volumes in men and women [28]. It seems to modulate cognitive functions depending on the hormonal status [75] and was associated with treatment resistance only in women [76]. Although a recent meta-analysis could not detect significant sex-related difference in the functional effect of the *COMT* rs4680 genotype [19], our findings suggest that sex should be taken into account in future studies focusing on the association between *COMT* polymorphisms and symptoms in schizophrenia.

In general, MAO-B genetic variants have been less extensively investigated than COMT genetic variants in patients with schizophrenia, despite the importance of MAO-B in degrading dopamine, particularly in the subcortical areas, where the majority of dopamine clearance occurs via dopamine transporter and MAO-B [70]. It was assumed that the A allele of the *MAO-B* rs1799836 was associated with higher MAO-B activity [42,77] and, therefore, may result in lower subcortical dopamine levels. However, some studies reported higher enzyme activity in G allele carriers [78,79], while our previous studies reported no differences in platelet MAO-B activity in carriers of the A or G allele [80,81,82,83,84], questioning the hypothesis of its functionality. In addition, *MAO-B* rs1799836 was not found to be a functional polymorphism in a meta-analysis [19]. The only significant finding in the present study with the *MAO-B* rs1799836 was the higher BNSS alogia subscale scores in A compared to G allele carriers, respectively. This is in partial disagreement with the findings of higher affective flattening in Mexican women with schizophrenia who were G carriers, although this significance was lost after Bonferroni correction, while no differences were detected in other negative symptoms, including anhedonia [44]. The discrepancies may arise from the differences in age, smoking status and ethnic differences, which all affect MAO-B activity [81,84], but also from the effects of other polymorphisms inside the *MAOB* gene. For example, participants in the latter study were 10 years younger than our patients [44].

The literature data on the *MAO-B* rs6651806 polymorphism are scarce. Its effect on the MAO-B activity is unknown, but the C allele carriers had three additional transcription factor-binding sites [45], which might change the enzyme activity. This polymorphism was also nominally associated with the cerebrospinal fluid 3-methoxy-4-hydroxyphenylglycol concentration in men with schizophrenia [46]. *MAO-B* rs6651806 was addressed in three clinical studies to date. It was not associated with schizophrenia in the Spanish population [85] but the *MAO-B* haplotype, which includes the rs6651806 polymorphism, was associated with obsessive-compulsive disorder [86]. The third trial of the MAO-B rs6651806 polymorphism reported a higher negative emotionality in the A allele carriers compared to C allele carriers, but the population included healthy participants, not separately analyzed by sex [45]. Our findings are in line with these results, since, in female students, high levels of social anhedonia were associated with a lower positive affect [87], while our female patients with schizophrenia who were A allele carriers had a greater social anhedonia level, and, more frequently, the presence of both social anhedonia and physical anhedonia. Male patients with schizophrenia who were A allele carriers had higher CAINS and BNSS total scores, greater CAINS recreational and expression scores, and BNSS anhedonia and alogia subscale scores than C allele carriers. These findings suggest that, although sex-specific, the A allele was associated with worse negative symptom pathology. In addition, male patients with schizophrenia with the *MAO-B* rs1799836–rs6651806 GC haplotype had lower CAINS expression and BNSS total and alogia subscale scores.

There is evidence that the gene variants, addressed in the present study, may affect efficacy and safety of medications used to treat different CNS disorders and may also be associated with certain biomarkers of the disease. For example, in patients with Parkinson’s disease, those with the *MAO-B* rs1799836 AA genotype were less likely to develop levodopa-induced dyskinesia than the AG or GG carriers [88]. In patients with schizophrenia, the *COMT* rs4680 genotype was associated with antipsychotic-induced dopamine hypersensitivity and resistance to antipsychotics [89]. Additionally, carriers of the *COMT* rs4680 A allele and *COMT* rs4680–rs4818 C-A haplotype had a better response to olanzapine treatment than carriers of the *COMT* rs4680 GG genotype and other *COMT* rs4680–rs4818 haplotypes [32]. In patients with dementia or mild cognitive impairment, cerebrospinal Aβ42 levels were decreased in participants with the GG compared to AG *COMT* rs4680 genotype, as well as in carriers of the A allele in *MAO-B* rs1799836 polymorphism [90].

Our findings further emphasize the role of MAO-B in negative symptoms of schizophrenia. This enzyme, apart from degrading dopamine, is also a key factor in determining the phenylethylamine concentrations in the brain [91,92]. Phenylethylamine was previously shown to promote energy and elevate mood while its main metabolite phenylacetic acid was decreased in biological fluids of schizophrenic patients [93]. More recently, phenylethylamine treatment restored anhedonia in mice [94]. Moreover, the inhibition of MAO-B is already a treatment target for Parkinson’s disease and major depression; those drugs have also been evaluated for their potential in the treatment of Alzheimer’s disease, Lewy Body diseases with dementia, amyotrophic lateral sclerosis, and Huntington’s disease [37]. Three selective MAO-B inhibitors approved for Parkinson’s disease are irreversible inhibitors selegiline and rasagiline and reversible inhibitor safinamide. Selegiline is also approved for the treatment of major depressive disorder. 

### Limitations

Social and physical anhedonia was assessed by self-rating scales, which may not cover all the multidimensional aspects of anhedonia [95]. Cognitive symptoms were not determined, which may also impact negative symptoms. Due to the cross-sectional design, the influence of dopamine-degrading enzyme polymorphisms on the dynamics of negative symptoms was not addressed. Therefore, our results represent only an association between *COMT* and *MAO-B* polymorphisms and negative symptoms, while the causation remains to be established. All patients received antipsychotics, which influence negative symptoms. Although the total antipsychotic dose, presented as chlorpromazine equivalent, did not correlate with the severity of negative symptoms measured with all the scales used in the present study, the influence of different antipsychotics cannot be ruled out. Finally, even though the influence of antipsychotics on anhedonia is not completely understood, some of these medications may actually worsen anhedonia, while others may alleviate anhedonia, such as aripiprazole or low-dose quetiapine [10].

The strengths of the present study are in both the genotype and haplotype analyses of the *COMT* rs4680 and rs4818 and *MAO-B* rs1799836 and rs6651806 polymorphisms. This is the first study of the relationship between *COMT* rs4818 or *MAO-B* rs6651806 polymorphisms and anhedonia, the first inclusion of ethnically homogenous Caucasian patients with schizophrenia, and the first detailed analysis of results, controlled for possible confounders (age of the patients, sex, smoking, alcohol consumption, history of drug abuse, history of suicide attempts and therapeutic dose of antipsychotic in chlorpromazine equivalents), in the evaluation of symptoms with the CAINS, BNSS, RPAS and RSAS, in patients with schizophrenia.

In female patients with schizophrenia, the presence of the G alleles of *the COMT* rs4680 and rs4818, as well as GG haplotype rs4818–rs4680, was associated with negative symptoms and anhedonia. In male patients with schizophrenia, the A allele of the *MAO-B* rs1799836 was related to the higher severity of alogia, while the A allele of the *MAO-B* rs6651806 was associated with higher negative symptom severity. These findings might suggest that higher dopamine degradation, associated with *COMT* and *MAO-B* genetic variants, is connected to a sex-specific greater severity of negative symptoms in schizophrenia patients. These results offer the possibility of using *COMT* and *MAO-B* genetic variants as biomarkers to predict negative symptoms in schizophrenia. Longitudinal studies are needed to establish the dynamics of those associations throughout different illness stages. In addition, our results extend the preliminary knowledge on the treatment potential of MAO-B and COMT inhibitors on specific symptoms of schizophrenia. In the first study, negative symptoms, particularly avolition, improved during treatment with the MAO-B inhibitor rasagiline in a group of predominantly male patients with schizophrenia [96]. The second study demonstrated the improvement in working memory with the COMT inhibitor tolcapone in healthy males who were carriers of the GG genotype of the *COMT* rs4680 [97]. Collectively, the present and the aforementioned studies encourage future research into add-on treatments with dopamine-metabolizing enzyme inhibitors in patients with schizophrenia, and suggest controlling patients for the *COMT* and *MAO-B* genotypes. Individualized treatment in schizophrenia, similar to other fields of medicine, is expected to be the most effective approach; taking various genotypes into account may help reach this goal. 

## 5. Conclusions

Anhedonia, one of the crucial negative symptoms in schizophrenia, is a complex construct, and its components are differently associated with the dopamine-metabolizing enzyme polymorphisms. Our findings suggest that higher dopamine degradation, related to *COMT* rs4680 and rs4818 variants, is associated with a sex-specific increase in severity of negative symptoms in schizophrenia patients. While the *MAO-B* rs1799836 was only associated with alogia in male patients with schizophrenia, the poorly studied *MAO-B* rs6651806 polymorphism was related to different negative symptoms, including anhedonia, in both sexes. These results suggest that the *COMT* and *MAO-B* genetic variants might be used as biomarkers of negative symptoms in schizophrenia.

## Figures and Tables

**Table 1:** Demographic and clinical data of patients with schizophrenia, depending on sex. The data are represented as total number and frequency or as median and interquartile range, while *p* values denoted in bold represent statistical significance.

		Male Patients (*n* = 178)		Female Patients (*n* = 124)		Statistics
Age		41 (31;50)		45 (38;53)		U = 9235.0; ***p* = 0.016**
BMI/kg		27.5 (24.3;30.9)		27.3 (23.6;32.4)		U = 11002.5; *p* = 0.964
First diagnosed/years		23 (19;30)		25 (19;33)		U = 10102.0; *p* = 0.210
Smoking	Non-smokers Smokers	76	42.7%	73	58.9%	χ^2^ = 7.649; df = 1; ***p* = 0.006**
		102	57.3%	51	41.1%	
Alcohol consumption	No	148	83.1%	114	91.9%	χ^2^ = 4.914; df = 1; ***p* = 0.027**
	Yes	30	16.9%	10	8.1%	
History of drug abuse	No	125	70.2%	111	89.5%	χ^2^ = 15.927; df = 1; ***p* < 0.001**
	Yes	53	29.8%	13	10.5%	
History of suicide attempts	No	139	78.1%	95	76.6%	χ^2^ = 0.091; df = 1; *p* = 0.762
	Yes	39	21.9%	29	23.4%	
Antipsychotic therapy	Typical	9	5.1%	8	6.6%	χ^2^ = 2.230; df = 2; *p* = 0.328
	Atypical	123	69.5%	74	61.2%	
	Combined	45	25.4%	39	32.2%	
Antipsychotic dose/mg/day ꭞ		550 (375;850)		538 (350;900)		U = 10,818.5; *p* = 0.771
GAF scores		50 (41;61)		51 (41;65)		U = 11,016.0; *p* = 0.979
PANSS scores		68 (60;79)		67 (59;81)		U = 10,700.5; *p* = 0.653
CDSS scores		1 (0;4)		2 (0;6)		U = 9755.5; *p* = 0.080
CAINS scores		23 (17;28)		21 (15;26)		U = 9904.0; *p* = 0.129
BNSS scores		29 (22;37)		27 (21;35)		U = 9938.5; *p* = 0.141
RPAS scores		19 (13;25)		17 (12;22)		U = 9302.0; ***p* = 0.020**
RSAS scores		12 (8;17)		12 (7;17)		U = 10613.5; *p* = 0.571
Physical anhedonia	No	141	79.2%	78	62.9%	χ^2^ = 9.755; df = 1; ***p* = 0.002**
	Yes	37	20.8%	46	37.1%	
Social anhedonia	No	151	84.8%	88	71.0%	χ^2^ = 8.509; df = 1; ***p* = 0.004**
	Yes	27	15.2%	36	36.0%	

ꭞ in chlorpromazine equivalents; BMI—body mass index; BNSS—The Brief Negative Symptom Scale; CAINS—Clinical Assessment Interview for Negative Symptoms; CDSS—The Calgary Depression Scale for Schizophrenia; GAF—Global Assessment of Functioning; PANSS—Positive and Negative Syndrome Scale; RPAS—Revised Physical Anhedonia Scale; RSAS—Revised Social Anhedonia Scale.

**Table 2:** The effect of demographic and clinical data on global functioning, severity of schizophrenia, depression, negative symptoms, and physical and social anhedonia in patients with schizophrenia, evaluated with a general linear model. *p* values in bold represent statistical significance.

Clinical Scale Model (adj. R^2^)	CAINS R^2^ = 0.185	BNSS R^2^ = 0.149	RPAS R^2^ = 0.095	RSAS R^2^ = 0.095
Predictors				
Age	F = 3.430; *p* = 0.065	F = 0.956; *p* = 0.329	F = 3.933; ***p* = 0.048**	F = 4.476; ***p* = 0.035**
Sex	F = 0.717; *p* = 0.398	F = 0.532; *p* = 0.467	F = 6.391; ***p* = 0.012**	F = 0.285; *p* = 0.594
Smoking	F = 1.483; *p* = 0.224	F = 2.034; *p* = 0.155	F = 2.752; *p* = 0.098	F = 4.307; ***p* = 0.039**
Alcohol consumption	F = 0.863; *p* = 0.354	F = 0.225; *p* = 0.635	F = 0.047; *p* = 0.828	F = 0.256; *p* = 0.613
History of drug abuse	F = 0.038; *p* = 0.845	F = 0.581; *p* = 0.447	F = 0.263; *p* = 0.609	F = 0.315; *p* = 0.575
History of suicide attempts	F = 1.319; *p* = 0.252	F = 1.213; *p* = 0.272	F = 0.328; *p* = 0.567	F = 0.263; *p* = 0.608
Antipsychotic dose ꭞ	F = 13.226; ***p* < 0.001**	F = 11.314; ***p* = 0.001**	F = 0.588; *p* = 0.444	F = 1.671; *p* = 0.197

ꭞ in chlorpromazine equivalents; BMI—body mass index; BNSS—The Brief Negative Symptom Scale; CAINS—Clinical Assessment Interview for Negative Symptoms; RPAS—Revised Physical Anhedonia Scale; RSAS—Revised Social Anhedonia Scale.

**Table 3:** Depressive symptoms, negative symptoms, and physical and social anhedonia depending on *COMT* rs4680 and COMT rs4818 polymorphisms, and their haplotypes, in male and female patients with schizophrenia. The data are denoted as median and interquartile range, while *p* values in bold represent statistical significance.

		CAINS	BNSS	RPAS	RSAS
**Male patients (*n* = 178)**					
*COMT* rs4680	AA	23 (18;29)	28 (23;36)	20 (13;27)	13 (8;17)
	AG	23 (16;28)	29 (20;37)	19 (14;25)	11 (8;16)
	GG	23 (17;28)	28 (23;35)	16 (12;22)	14 (8;18)
Statistics		H = 0.350; df = 2; *p* = 0.839	H = 0.073; df = 2; *p* = 0.964	H = 3.433; df = 2; *p* = 0.180	H = 1.851; df = 2; *p* = 0.396
*COMT* rs4680	A	23 (17;28)	29 (23;36)	20 (14;26)	12 (8;16)
	G	23 (17;28)	29 (21;37)	17 (17;23)	12 (8;17)
Statistics		U = 15,428.5; *p* = 0.734	U = 15,497.5; *p* = 0.788	U = 14,072.5; *p* = 0.082	U = 15,491.5; *p* = 0.783
*COMT* rs4818	CC	23 (18;28)	28 (23;36)	20 (13;26)	12 (8;17)
	CG	22 (16;28)	29 (20;37)	19 (13;25)	12 (7;16)
	GG	23 (19;28)	29 (23;34)	17 (13;20)	14 (8;18)
Statistics		H = 0.559; df = 2; *p* = 0.756	H = 0.110; df = 2; *p* = 0.946	H = 1.896; df = 2; *p* = 0.387	H = 1.168; df = 2; *p* = 0.558
*COMT* rs4818	C	23 (17;28)	29 (22;37)	19 (13;25)	12 (8;17)
	G	23 (16;28)	29 (22;37)	18 (13;23)	13 (8;17)
Statistics		U = 14,431.5; *p* = 0.744	U = 14,495.5; *p* = 0.796	U = 13,528.5; *p* = 0.196	U = 14,669.5; *p* = 0.942
*COMT* rs4818-rs4680	CA	23 (17;28)	29 (23;36)	20 (14;26)	12 (8;16)
	GG	23 (16;28)	29 (22;37)	18 (13;23)	13 (8;18)
	CG	23 (17;28)	28 (20;39)	17 (12;24)	12 (8;18)
Statistics		H = 0.164; df = 2; *p* = 0.921	H = 0.089; df = 2; *p* = 0.956	H = 3.027; df = 2; *p* = 0.220	H = 0.255; df = 2; *p* = 0.880
**Female patients (*n* = 124)**					
*COMT* rs4680	AA	19 (15;23)	14 (10;21)	14 (10;21)	11 (6;14)
	AG	21 (15;26)	18 (12;22)	18 (12;22)	12 (7;16)
	GG	24 (18;28)	18 (11;23)	18 (11;23)	13 (7;17)
Statistics		H = 5.573; df = 2; *p* = 0.056	H = 0.595; df = 2; *p* = 0.743	H = 0.595; df = 2; *p* = 0.743	H = 0.567; df = 2; *p* = 0.753
*COMT* rs4680	A	20 (15;24)	25 (19;33)	17 (17;21)	12 (7;15)
	G	22 (16;28)	27 (22;37)	18 (12;22)	13 (7;17)
Statistics		U = 6245.0; ***p* = 0.015**	U = 6674.0; *p* = 0.094	U = 7215.0; *p* = 0.475	U = 7199; *p* = 0.458
*COMT* rs4818	CC	19 (15;23)	24 (18;32)	17 (12;21)	10 (6;14)
	CG	22 (15;27)	27 (20;38)	18 (13;22)	13 (8;17)
	GG	24 (18;29)	27 (24;37)	17 (10;23)	13 (7;17)
Statistics		H = 6.838; df = 2; ***p* = 0.033**	H = 2.650; df = 2; *p* = 0.266	H = 0.223; df = 2; *p* = 0.895	H = 2.789; df = 2; *p* = 0.248
*COMT* rs4818	C	20 (15;24)	27 (19;33)	17 (12;21)	11 (7;15)
	G	23 (16;28)	27 (22;37)	18 (11;22)	13 (7;17)
Statistics		U = 6063.0; ***p* = 0.006**	U = 6649.0; *p* = 0.093	U = 7388.0; *p* = 0.719	U = 6732.0; *p* = 0.126
*COMT* rs4818-rs4680	CA	20 (15;24)	25 (19;33)	17 (12;21)	12 (7;15)
	GG	23 (16;28)	27 (22;37)	18 (11;22)	13 (7;17)
	CG	22 (15;25)	29 (16;33)	19 (13;22)	11 (6;14)
Statistics		H = 7.579; df = 2; ***p* = 0.023**	H = 3.108; df = 2; *p* = 0.211	H = 0.648; df = 2; *p* = 0.723	H = 3.065; df = 2; *p* = 0.216

BNSS—The Brief Negative Symptom Scale; CAINS—Clinical Assessment Interview for Negative Symptoms; COMT—catechol-O-methyl transferase; RPAS—Revised Physical Anhedonia Scale; RSAS—Revised Social Anhedonia Scale.

**Table 4:** Global functioning, severity of schizophrenia, depression, negative symptoms and physical and social anhedonia depending on *MAO-B* rs1799836 and *MAO-B* rs6651806 polymorphisms, and their haplotypes, in male and female patients with schizophrenia. The data are denoted as median and interquartile range, while *p* values in bold represent statistical significance.

		CAINS	BNSS	RPAS	RSAS
**Male patients (*n* = 178)**					
*MAO-B* rs1799836	A	24 (18;8)	31 (23;37)	19 (13;24)	13 (8;17)
	G	22 (17;28)	27 (21;35)	18 (12;23)	12 (8;17)
Statistics		U = 2982.5; *p* = 0.137	U = 2943.5; *p* = 0.107	U = 3259.0; *p* = 0.553	U = 3296.5; *p* = 0.637
*MAO-B* rs6651806	A	24 (19;28)	29 (24;37)	18 (13;24)	13 (8;17)
	C	20 (13;28)	24 (17;35)	20 (13;23)	11 (8;17)
Statistics		U = 2212.5; ***p* = 0.036**	U = 2105.0; ***p* = 0.013**	U = 2742.5; *p* = 0.846	U = 2627.5; *p* = 0.544
*MAO-B* rs1799836-rs6651806	AA	24 (19;29)	32 (24;37)	19 (13;26)	13 (8;17)
	GC	19 (13;25)	24 (17;34)	20 (13;25)	11 (8;17)
	GA	23 (19;28)	28 (25;35)	17 (11;23)	12 (8;17)
		H = 5.720; df = 2; *p* = 0.057	H = 7.229; df = 2; ***p* = 0.027**	H = 1.558; df = 2; *p* = 0.459	H = 0.576; df = 2; *p* = 0.750
**Female patients (*n* = 124)**					
*MAO-B* rs1799836	AA	23 (14;26)	28 (23;36)	17 (10;23)	14 (7;20)
	GA	22 (16;28)	27 (22;37)	18 (13;22)	13 (7;17)
	GG	20 (15;24)	23 (19;31)	17 (12;20)	11 (6;14)
Statistics		H = 1.771; df = 2; *p* = 0.413	H = 1.865; df = 2; *p* = 0.394	H = 1.173; df = 2; *p* = 0.556	H = 2.592; df = 2; *p* = 0.274
*MAO-B* rs1799836	A	22 (15;26)	28 (22;36)	18 (11;22)	13 (7;17)
	G	21 (15;26)	25 (20;33)	17 (12;21)	11 (7;16)
Statistics		U = 6662.5; *p* = 0.328	U = 6620.5; *p* = 0.291	U = 6880.5; *p* = 0.567	U = 6381.5; *p* = 0.133
*MAO-B* rs6651806	AA	21 (15;25)	28 (21;33)	18 (12;22)	14 (7;19)
	AC	21 (16;29)	26 (20;40)	17 (11;22)	11 (6;14)
	CC	21 (14;27)	25 (21;32)	18 (13;19)	9 (5;14)
Statistics		H = 0.533; df = 2; *p* = 0.766	H = 0.401; df = 2; *p* = 0.818	H = 0.848; df = 2; *p* = 0.654	H = 4.331; df = 2; *p* = 0.115
*MAO-B* rs6651806	A	21 (16;25)	27 (21;34)	18 (12;22)	13 (7;17)
	C	21 (15;27)	26 (21;37)	17 (12;20)	11 (6;14)
Statistics		U = 6153.5; *p* = 0.946	U = 6107.5; *p* = 0.872	U = 5719.5; *p* = 0.347	U = 5115.5; ***p* = 0.031**
*MAO-B* rs1799836-rs6651806	AA	22 (15;26)	28 (22;36)	18 (11;22)	13 (7;17)
	GC	22 (15;27)	26 (21;37)	17 (11;20)	11 (6;14)
	GA	20 (17;24)	25 (19;32)	18 (13;22)	13 (9;17)
Statistics		H = 1.197; df = 2; *p* = 0.550	H = 1.228; df = 2; *p* = 0.541	H = 0.898; df = 2; *p* = 0.638	H = 4.565; df = 2; *p* = 0.102

BNSS—The Brief Negative Symptom Scale; CAINS—Clinical Assessment Interview for Negative Symptoms; MAO-B—monoamine oxidase B; RPAS—Revised Physical Anhedonia Scale; RSAS—Revised Social Anhedonia Scale.

## Data Availability

The data that support the findings of this study are available on request from the corresponding author (N.P.).

## Supplementary Materials

The following are available online at https://www.mdpi.com/article/10.3390/cimb43020045/s1.
