# Supplementary material for: The Associations between COMT and MAO-B Genetic Variants with Negative Symptoms in Patients with Schizophrenia
Source: Curr Issues Mol Biol. 2021 Jul 8;43(2):618–36. doi: 10.3390/cimb43020045 (PMC8928957; doi:10.3390/cimb43020045)
Supplement: Supplementary file 1 [file cimb-43-00045-s001.zip › cimb-1260707-supplementary.pdf]

current issues in molecular biology

MDPI

Article

The Associations between COMT and MAO-B Genetic Variants with Negative Symptoms in Patients with Schizophrenia

Zoran Madzarac [1], Lucija Tudor [2], Marina Sagud [1,3], Gordana Nedic Erjavec [2], Alma Mihaljevic Peles [1,3] and Nela Pivac [2,*]

[1] Department of Psychiatry and Psychological Medicine, University Hospital Centre Zagreb, 10 000 Zagreb, Croatia; zoranmadzarac@gmail.com (Z.M.); marinasagud@mail.com (M.S.); apeles@mef.hr (A.M.P.)
[2] Ruder Boskovic Institute, 10 000 Zagreb, Croatia; lucija.tudor@irb.hr (L.T.); gnedic@irb.hr (G.N.E.)
[3] School of Medicine, University of Zagreb, 10 000 Zagreb, Croatia
[*] Correspondence: npivac@irb.hr; Tel.: +385-915-371-810

Supplementary Table S1. The distribution of COMT rs4680 and rs4818 and MAO-B rs1799836 and rs6651806 genotypes and alleles, as well as their haplotypes, in male and female patients with schizophrenia.

		Male Patients (N=178)	Female Patients (N=124)	Statistics
COMT rs4680	AA	28.7%	21.8%	χ^2=4.421; df=2; p=0.110
	AG	50.0%	46.8%	
	GG	21.3%	31.5%	
	A	53.7%	45.2%	χ^2=4.215; df=1; **p=0.040**
	G	46.3%	54.8%	
COMT rs4818	CC	38.8%	33.1%	χ^2=4.857; df=2; p=0.088
	CG	48.9%	45.2%	
	GG	12.4%	21.8%	
	C	63.2%	55.6%	χ^2=3.481; df=2; p=0.062
	G	36.8%	44.4%	
COMT rs4818-rs4680	CA	53.4%	45.2%	χ^2=4.289; df=2; p=0.117
	GG	36.5%	44.4%	
	CG	9.8%	10.5%	
	GA	0.3%	0.0%	
MAO-B rs1799836	AA	-	26.7%	-
	AG	-	50.8%	
	GG	-	22.5%	
	A	51.2%	52.1%	χ^2=0.030; df=1; p=0.860
	G	48.8%	47.9%	
MAO-B rs6651806	AA	-	49.2%	-
	AC	-	39.2%	
	CC	-	11.7%	
	A	71.7%	68.8%	χ^2=3.481; df=1; p=0.062
	C	28.3%	31.3%	
MAO-B rs1799836- rs6651806	AA	50.6%	51.6%	χ^2=1.947; df=2; p=0.378
	GC	27.2%	31.9%	
	GA	21.1%	16.5%	
	AC	1.1%	0.0%	

COMT – catechol-O-methyl transferase; MAO-B – monoamine oxidase B.

Supplementary Table S2. Severity of negative symptoms evaluated with CAINS subscales for socialization, vocational, recreation and expression depending on the COMT rs4680 and COMT rs4818 polymorphisms and their haplotype, in male and female patients with schizophrenia. The data are denoted as median and interquartile range, while p values in bold represent statistical significance.

		CAINS Socialization	CAINS Vocational	CAINS Recreation	CAINS Expression
**Male patients (N=178)**					
COMT rs4680	AA	6 (4;9)	4 (7;3)	2 (4;7)	7 (5;9)
	AG	6 (4;8)	3 (7;3)	2 (4;7)	7 (5;9)
	GG	6 (5;8)	3 (7;3)	2 (4;7)	7 (5;9)
Statistics		H=0.230; df=2; p=0.891	H=2.401; df=2; p=0.301	H=0.063; df=2; p=0.969	H=0.248; df=2; p=0.884
COMT rs4680	A	6 (4;8)	6 (4;7)	3 (2;4)	7 (5;9)
	G	6 (4;8)	5 (3;7)	3 (2;4)	7 (5;9)
Statistics		U=15658.5; p=0.918	U=14911.5; p=0.378	U=15571.5; p=0.844	U=15666.5; p=0.925
COMT rs4818	CC	6 (4;8)	6 (4;7)	3 (2;4)	7 (5;9)
	CG	6 (4;8)	5 (3;7)	3 (2;4)	6 (5;8)
	GG	7 (5;8)	5 (3;7)	3 (2;4)	8 (5;9)
Statistics		H=0.379; df=2; p=0.827	H=1.006; df=2; p=0.605	H=0.041; df=2; p=0.980	H=1.598; df=2; p=0.450
COMT rs4818	C	6 (4;8)	6 (4;7)	3 (2;4)	7 (5;9)
	G	6 (4;8)	5 (3;7)	3 (2;4)	7 (5;9)
Statistics		U=14677.5; p=0.949	U=14004.5; p=0.429	U=14635.5; p=0.911	U=14634.4; p=0.912
COMT rs4818-rs4680	CA	6 (4;8)	6 (4;7)	3 (2;4)	7 (5;9)
	GG	6 (4;8)	5 (3;7)	3 (2;4)	7 (5;9)
	CG	6 (5;8)	6 (3;7)	3 (2;4)	6 (4;9)
Statistics		H=0.054; df=2; p=0.973	H=0.824; df=2; p=0.662	H=0.048; df=2; p=0.976	H=0.024; df=2; p=0.988
**Female patients (N=124)**					
COMT rs4680	AA	5 (2;6)	4 (3;7)	2 (1;3)	6 (4;8)
	AG	5 (2;7)	5 (3;7)	3 (2;4)	6 (4;9)
	GG	6 (4;8)	6 (4;8)	3 (2;4)	6 (4;10)
Statistics		H=6.719; df=2; **p=0.035**	H=3.830; df=2; p=0.147	H=4.901; df=2; p=0.086	H=0.323; df=2; p=0.851
COMT rs4680	A	5 (3;6)	5 (3;7)	2 (2;4)	6 (4;8)
	G	6 (4;7)	6 (4;8)	3 (2;4)	6 (4;9)
Statistics		U=6144.0; **p=0.008**	U=6499.0; **p=0.044**	U=6384.0; **p=0.025**	U=7342; p=0.624
COMT rs4818	CC	5 (3;6)	4 (3;6)	2 (1;3)	6 (4;8)
	CG	5 (4;7)	6 (4;8)	3 (2;4)	6 (4;9)
	GG	7 (5;8)	6 (4;8)	4 (2;4)	6 (4;8)
Statistics		H=8.813; df=2; **p=0.012**	H=7.835; df=2; **p=0.020**	H=4.817; df=2; p=0.090	H=0.433; df=2; p=0.805
COMT rs4818	C	5 (3;6)	5 (3;7)	2 (2;4)	6 (4;8)
	G	6 (4;8)	6 (4;8)	3 (2;4)	6 (4;9)
Statistics		U=5931.0; **p=0.003**	U=6076.0; **p=0.006**	U=6349.0; **p=0.024**	U=7274.0; p=0.571
COMT rs4818-rs4680	CA	5 (3;6)	5 (3;7)	2 (2;4)	6 (4;8)
	GG	6 (4;8)	6 (4;8)	3 (2;4)	6 (4;9)
	CG	5 (4;6)	5 (2;6)	3 (2;4)	6 (4;10)
Statistics		H=9.026; df=2; **p=0.011**	H=7.564; df=2; **p=0.023**	H=5.585; df=2; p=0.061	H=0.324; df=2; p=0.850

CAINS—Clinical Assessment Interview for Negative Symptoms; COMT—catechol-O-methyl transferase.

Supplementary Table S3. Severity of negative symptoms evaluated with BNSS subscales for anhedonia, asocialization, avolition, blunted effect and alogia, depending on the COMT rs4680 and COMT rs4818 polymorphisms and their haplotype, in male and female patients with schizophrenia. The data are denoted as median and interquartile range, while p values in bold represent statistical significance.

		BNSS Anhedonia	BNSS Asocialization	BNSS Avolition	BNSS Blunted Effect	BNSS Alogia
**Male patients (N=178)**						
COMT rs4680	AA	6 (5;8)	4 (3;5)	6 (4;8)	8 (6;11)	2 (1;4)
	AG	6 (4;9)	4 (2;5)	5 (3;7)	8 (6;11)	3 (0;4)
	GG	6 (4;9)	4 (3;5)	6 (3;8)	8 (6;11)	2 (1;4)
Statistics		H=0.460; df=2; p=0.794	H=0.156; df=2; p=0.925	H=2.223; df=2; p=0.329	H=0.578; df=2; p=0.749	H=0.907; df=2; p=0.635
COMT rs4680	A	6 (4;9)	4 (3;5)	6 (4;8)	8 (6;11)	2 (1;4)
	G	6 (4;9)	4 (3;5)	5 (3;7)	8 (6;11)	2 (1;4)
Statistics		U=15640.5; p=0.903	U=15620.5; p=0.884	U=14588.5; p=0.224	U=15744.5; p=0.989	U=15502.5; p=0.789
COMT rs4818	CC	6 (5;8)	4 (3;5)	6 (4;8)	8 (5;11)	2 (1;4)
	CG	6 (4;10)	4 (2;5)	5 (2;7)	8 (6;11)	2 (1;4)
	GG	6 (4;9)	4 (3;4)	6 (3;7)	8 (6;11)	2 (1;4)
Statistics		H=0.332; df=2; p=0.847	H=0.244; df=2; p=0.885	H=1.093; df=2; p=0.386	H=0.251; df=2; p=0.882	H=0.706; df=2; p=0.703
COMT rs4818	C	6 (4;9)	4 (2;5)	6 (4;8)	8 (6;11)	2 (1;4)
	G	6 (4;10)	4 (3;5)	5 (3;7)	8 (6;11)	2 (1;4)
Statistics		U=14583.5; p=0.869	U=14578.5; p=0.861	U=13614.5; p=0.227	U=14683.5; p=0.954	U=14727.5; p=0.991
COMT rs4818-rs4680	CA	6 (4;9)	4 (3;5)	6 (4;8)	8 (6;11)	2 (1;4)
	GG	6 (4;9)	4 (3;5)	5 (3;7)	8 (6;11)	2 (1;4)
	CG	6 (4;9)	4 (2;5)	6 (3;8)	8 (6;12)	3 (0;4)
Statistics		H=0.072; df=2; p=0.965	H=0.162; df=2; p=0.922	H=1.696; df=2; p=0.428	H=0.053; df=2; p=0.974	H=0.187; df=2; p=0.911
**Female patients (N=124)**						
COMT rs4680	AA	5 (3;7)	3 (2;4)	4 (4;7)	8 (6;10)	2 (0;4)
	AG	5 (3;9)	3 (2;4)	5 (3;6)	8 (5;11)	2 (0;4)
	GG	8 (5;10)	4 (2;5)	6 (4;8)	8 (6;12)	2 (0;5)
Statistics		H=5.286; df=2; p=0.071	H=4.295; df=2; p=0.117	H=2.688; df=2; p=0.261	H=0.295; df=2; p=0.863	H=1.146; df=2; p=0.564
COMT rs4680	A	5 (3;8)	3 (2;4)	4 (3;7)	8 (6;11)	2 (0;4)
	G	7 (4;9)	4 (2;4)	6 (3;7)	8 (6;12)	2 (0;4)
Statistics		U=6336.0; **p=0.022**	U=6676.0; p=0.081	U=6874.0; p=0.183	U=7303.0; p=0.576	U=7075.0; p=0.323
COMT rs4818	CC	5 (3;7)	3 (2;4)	4 (2;6)	8 (6;10)	2 (0;3)
	CG	7 (4;9)	3 (2;4)	5 (3;8)	8 (6;12)	2 (0;4)
	GG	8 (4;10)	4 (2;5)	6 (3;8)	8 (6;12)	2 (0;4)
Statistics		H=6.653; df=2; **p=0.034**	H=3.406; df=2; p=0.182	H=2.656; df=2; p=0.265	H=1.230; df=2; p=0.541	H=0.115; df=2; p=0.944

COMT rs4818	C	5 (3;8)	3 (2;4)	5 (3;7)	8 (6;11)	2 (0;4)
	G	7 (4;10)	4 (2;4)	6 (3;8)	8 (6;12)	2 (0;4)
Statistics		U=6119.0; **p=0.008**	U=6700.0; p=0.098	U=6752.0; p=0.132	U=7149.0; p=0.430	U=7515.0; p=0.891
COMT rs4818-rs4680	CA	5 (3;8)	3 (2;4)	4 (3;7)	8 (6;11)	2 (0;4)
	GG	7 (4;10)	4 (2;4)	6 (3;8)	8 (6;12)	2 (0;4)
	CG	6 (3;9)	4 (2;6)	6 (2;7)	8 (4;11)	2 (2;5)
Statistics		H=7.0421; df=2; **p=0.030**	H=3.206; df=2; p=0.201	H=2.305; df=2; p=0.316	H=0.641; df=2; p=0.726	H=2.260; df=2; p=0.323

BNSS—The Brief Negative Symptom Scale; COMT—catechol-O-methyl transferase.

Supplementary Table S4. The distribution of COMT rs4680 and COMT rs4818 genotypes, alleles and haplotypes depending on the presence of physical and social anhedonia in male and female patients with schizophrenia. P values in bold represent statistical significance.

		**Physical Anhedonia**		**Social Anhedonia**	
**Male patients (N=178)**					
		**No (N=141)**	**Yes (N=37)**	**No (151)**	**Yes (N=27)**
COMT rs4680	AA	28.4%	29.7%	29.1%	25.9%
	AG	49.6%	51.4%	49.7%	51.9%
	GG	22.0%	18.9%	21.2%	22.2%
Statistics		χ^2=0.165; df=2; p=0.912		χ^2=1.116; df=2; p=0.944	
COMT rs4680	A	53.2%	55.4%	54.0%	51.9%
	G	46.8%	44.6%	46.0%	48.1%
Statistics		χ^2=0.116; df=1; p=0.734		χ^2=0.083; df=1; p=0.773	
COMT rs4818	CC	39.0%	37.8%	38.4%	40.7%
	CG	48.2%	51.4%	49.0%	48.1%
	GG	12.8%	10.8%	12.6%	11.1%
Statistics		χ^2=0.160; df=2; p=0.923		χ^2=0.076; df=2; p=0.963	
COMT rs4818	C	63.1%	63.5%	62.9%	64.8%
	G	36.9%	36.5%	37.1%	35.2%
Statistics		χ^2=0.004; df=1; p=0.950		χ^2=0.071; df=1; p=0.790	
COMT rs4818-rs4680	CA	53.0%	55.4%	53.8%	51.9%
	GG	36.7%	36.5%	36.9%	35.2%
	GC	10.3%	8.1%	9.3%	13.0%
Statistics		χ^2=0.353; df=2; p=0.838		χ^2=0.691; df=2; p=0.708	
**Female patients (N=124)**					
		**No (N=78)**	**Yes (N=46)**	**No (N=88)**	**Yes (N=36)**
COMT rs4680	AA	21.8%	21.7%	25.0%	13.9%
	AG	46.2%	47.8%	46.6%	47.2%
	GG	32.1%	30.4%	28.4%	38.9%
Statistics		χ^2=0.041; df=2; p=0.980		χ^2=2.343; df=2; p=0.310	
COMT rs4680	A	44.9%	45.7%	48.3%	37.5%
	G	55.1%	54.3%	51.7%	62.5%
Statistics		χ^2=0.014; df=1; p=0.905		χ^2=2.404; df=1; p=0.121	
COMT rs4818	CC	33.3%	32.6%	38.6%	19.4%
	CG	43.6%	47.8%	42.0%	52.8%
	GG	23.1%	19.6%	19.3%	27.8%
Statistics		χ^2=0.283; df=2; p=0.868		χ^2=4.337; df=2; p=0.114	
COMT rs4818	C	55.1%	56.5%	59.7%	45.8%

	G	44.9%	43.5%	40.3%	54.2%
Statistics		χ^2=0.046; df=1; p=0.831		χ^2=3.957; df=1; **p=0.047**	
COMT rs4818-rs4680	CA	44.9%	45.7%	48.3%	37.5%
	GG	44.9%	43.5%	40.3%	54.2%
	GC	10.3%	10.9%	11.4%	8.3%
Statistics		χ^2=0.054; df=2; p=0.973		χ^2=3.968; df=2; p=0.138	

COMT—catechol-O-methyl transferase.

Supplementary Table S5. Severity of negative symptoms evaluated with CAINS subscales for socialization, vocational, recreation and expression depending on the MAO-B rs1799836 and MAO-B rs6651806 polymorphisms, and their haplotype, in male and female patients with schizophrenia. The data are denoted as median and interquartile range, while p values in bold represent statistical significance.

		CAINS Socialization	CAINS Vocational	CAINS Recreation	CAINS Expression
**Male patients (N=178)**					
MAO-B rs1799836	A	6 (4;8)	5 (4;7)	3 (2;4)	7 (5;9)
	G	6 (4;8)	6 (3;7)	3 (2;4)	6 (4;8)
Statistics		U=3223.5; p=0.477	U=3416.0; p=0.931	U=3159.0; p=0.348	U=2866.5; p=0.061
MAO-B rs6651806	A	6 (4;8)	5 (4;7)	3 (2;4)	7 (5;9)
	C	6 (3;9)	4 (3;7)	2 (2;4)	5 (4;8)
Statistics		U=2721.0; p=0.785	U=2411.5; p=0.163	U=2223.0; **p=0.035**	U=2132.0; **p=0.017**
MAO-B rs1799836-rs6651806	AA	6 (4;8)	5 (4;7)	3 (2;4)	7 (5;9)
	GC	6 (3;8)	6 (3;7)	2 (1;4)	5 (4;8)
	GA	6 (4;8)	6 (4;7)	3 (2;4)	7 (5;8)
Statistics		H=1.506; df=2; p=0.471	H=1.143; df=2; p=0.565	H=5.226; df=2; p=0.073	H=6.308; df=2; **p=0.043**
**Female patients (N=124)**					
MAO-B rs1799836	AA	6 (4;8)	6 (4;7)	3 (2;4)	6 (4;8)
	AG	5 (4;7)	6 (4;7)	3 (2;4)	7 (5;9)
	GG	5 (3;6)	4 (3;8)	3 (1;4)	6 (4;8)
Statistics		H=2.287; df=2; p=0.319	H=1.194; df=2; p=0.551	H=0.960; df=2; p=0.619	H=1.688; df=2; p=0.430
MAO-B rs1799836	A	6 (4;7)	6 (4;7)	3 (2;4)	6 (4;9)
	G	5 (4;6)	5 (3;7)	3 (2;4)	6 (4;9)
Statistics		U=6404.5; p=0.142	U=7080.5; p=0.840	U=6724.5; p=0.379	U=7102.5; p=0.874
MAO-B rs6651806	AA	6 (3;7)	6 (3;7)	3 (2;4)	6 (4;8)
	AC	5 (4;7)	6 (4;7)	3 (2;4)	6 (4;10)
	CC	6 (3;6)	4 (3;6)	2 (1;4)	7 (4;9)
Statistics		H=0.333; df=2; p=0.847	H=1.522; df=2; p=0.467	H=2.501; df=2; p=0.286	H=0.555; df=2; p=0.758
MAO-B rs6651806	A	5 (4;7)	6 (4;7)	3 (2;4)	6 (4;8)
	C	5 (4;7)	5 (3;7)	3 (2;4)	7 (4;9)
Statistics		U=6037.5; p=0.762	U=5872.5; p=0.523	U=6129.5; p=0.905	U=5802.5; p=0.437
MAO-B rs1799836-rs6651806	AA	6 (4;7)	6 (4;7)	3 (2;4)	6 (4;9)
	GC	5 (4;7)	5 (3;7)	3 (2;4)	6 (4;9)
	GA	5 (3;6)	6 (3;8)	2 (1;4)	6 (4;8)
Statistics		H=1.846; df=2; p=0.397	H=0.438; df=2; p=0.803	H=1.742; df=2; p=0.419	H=0.704; df=2; p=0.703

CAINS—Clinical Assessment Interview for Negative Symptoms; MAO-B—monoamine oxidase B.

Supplementary Table S6. Severity of negative symptoms evaluated with BNSS subscales for anhedonia, asocialization, avolition, blunted effect and alogia, depending on the MAO-B rs1799836 and MAO-B rs6651806 polymorphisms, and their haplotype, in male and female patients with schizophrenia. The data are denoted as median and interquartile range, while p values in bold represent statistical significance.

		BNSS Anhedonia	BNSS Asocialization	BNSS Avolition	BNSS Blunted Effect	BNSS Alogia
**Male patients (N=178)**						
MAO-B rs1799836	A	6 (5;9)	4 (3;5)	6 (4;8)	8 (7;11)	2 (2;4)
	G	6 (3;9)	4 (2;5)	6 (4;7)	7 (5;11)	2 (0;4)
Statistics		U=3140.0; p=0.511	U=3243.0; p=0.505	U=3382.0; p=0.844	U=3062.5; p=0.218	U=2765.0; **p=0.026**
MAO-B rs6651806	A	6 (5;9)	4 (3;5)	6 (4;7)	8 (6;11)	2 (2;4)
	C	5 (3;9)	4 (2;5)	5 (2;8)	7 (5;11)	1 (0;4)
Statistics		U=2208.5; **p=0.034**	U=2413.0; p=0.155	U=2587.0; p=0.449	U=2360.5; p=0.117	U=1949.5; **p=0.002**
MAO-B rs1799836-rs6651806	AA	6 (5;9)	4 (3;5)	6 (4;8)	8 (7;11)	2 (2;4)
	GC	5 (3;8)	4 (2;4)	5 (3;8)	7 (5;10)	1 (0;3)
	GA	7 (5;9)	4 (3;5)	6 (4;7)	9 (6;11)	2 (1;5)
Statistics		H=4.963; df=2; p=0.084	H=2.358; df=2; p=0.308	H=0.627; df=2; p=0.731	H=3.861; df=2; p=0.145	H=9.047; df=2; **p=0.011**
**Female patients (N=124)**						
MAO-B rs1799836	AA	7 (5;9)	4 (2;4)	6 (3;7)	9 (6;10)	2 (0;4)
	AG	6 (4;9)	4 (2;4)	6 (4;7)	8 (6;12)	2 (1;4)
	GG	5 (3;9)	3 (2;4)	4 (2;6)	8 (5;11)	2 (0;3)
Statistics		H=0.779; df=2; p=0.677	H=1.744; df=2; p=0.418	H=2.951; df=2; p=0.229	H=0.325; df=2; p=0.850	H=3.266; df=2; p=0.195
MAO-B rs1799836	A	7 (4;9)	4 (2;4)	6 (4;7)	8 (6;11)	2 (0;4)
	G	5 (3;9)	3 (2;4)	5 (3;7)	8 (6;11)	2 (0;4)
Statistics		U=6744.5; p=0.406	U=6648.5; p=0.297	U=6693.5; p=0.354	U=7231.5; p=0.917	U=7180.5; p=0.989
MAO-B rs6651806	AA	6 (3;8)	4 (2;4)	5 (4;8)	8 (6;10)	2 (0;4)
	AC	7 (4;10)	4 (2;4)	5 (4;7)	8 (5;12)	2 (0;4)
	CC	5 (3;9)	2 (2;4)	4 (2;6)	9 (9;11)	2 (1;4)
Statistics		H=3.331; df=2; p=0.189	H=1.414; df=2; p=0.493	H=2.754; df=2; p=0.252	H=1.314; df=2; p=0.519	H=0.068; df=2; p=0.967
MAO-B rs6651806	A	6 (4;8)	4 (2;4)	5 (4;7)	8 (6;11)	2 (0;4)
	C	6 (3;9)	3 (2;4)	5 (3;7)	8 (6;12)	2 (0;4)
Statistics		U=5937.5; p=0.613	U=5665.5; p=0.276	U=5745.5; p=0.371	U=5612.5; p=0.247	U=6062.5; p=0.797
MAO-B rs1799836-rs6651806	AA	7 (4;9)	4 (2;4)	6 (3;7)	8 (6;11)	2 (0;4)
	GC	7 (3;10)	4 (2;4)	5 (3;7)	8 (6;12)	2 (0;4)
	GA	5 (3;7)	3 (2;4)	5 (4;7)	8 (6;10)	2 (0;4)
Statistics		H=2.725; df=2; p=0.256	H=0.595; df=2; p=0.743	H=0.6337; df=2; p=0.727	H=2.918; df=2; p=0.232	H=0.210 df=2; p=0.900

BNSS—The Brief Negative Symptom Scale; MAO-B—monoamine oxidase B.

Supplementary Table S7. The distribution of MAO-B rs1799836 and MAO-B rs6651806 genotypes, alleles and haplotypes depending on the presence of physical and social anhedonia in male and female patients with schizophrenia. P values in bold represent statistical significance.

		Physical Anhedonia		Social Anhedonia	
**Male Patients (N=178)**		**No (N=141)**	**Yes (N=37)**	**No (151)**	**Yes (N=27)**
MAO-B rs1799836	A	52.2%	46.9%	50.3%	56.5%
	G	47.8%	53.1%	49.7%	43.5%
Statistics		χ^2=0.297; df=1; p=0.585		χ^2=0.302; df=1; p=0.583	
MAO-B rs6651806	A	69.4%	81.3%	71.3%	73.9%
	C	30.6%	18.8%	28.7%	26.1%
Statistics		χ^2=1.786; df=1; p=0.181		χ^2=0.065; df=1; p=0.798	
MAO-B rs1799836 - rs6651806	AA	52.2%	48.6%	50.3%	57.7%
	GC	29.0%	21.6%	28.2%	23.1%
	GA	18.8%	29.7%	21.5%	19.2%
Statistics		χ^2=2.284; df=2; p=0.319		χ^2=0.497; df=2; p=0.780	
**Female patients (N=124)**		**No (N=78)**	**Yes (N=46)**	**No (N=88)**	**Yes (N=36)**
MAO-B rs1799836	AA	22.7%	33.3%	23.8%	33.3%
	GA	50.7%	51.1%	50.0%	52.8%
	GG	26.7%	15.6%	26.2%	13.9%
Statistics		χ^2=2.744; df=2; p=0.254		χ^2=2.590; df=2; p=0.274	
MAO-B rs1799836	A	48.0%	58.9%	48.8%	59.7%
	G	52.0%	41.1%	51.2%	40.3%
Statistics		χ^2=2.672; df=1; p=0.102		χ^2=2.405; df=1; p=0.121	
MAO-B rs6651806	AA	44.0%	57.8%	42.9%	63.9%
	AC	40.0%	37.8%	44.0%	27.8%
	CC	16.0%	4.4%	13.1%	8.3%
Statistics		χ^2=4.340; df=2; p=0.114		χ^2=4.460; df=2; p=0.108	
MAO-B rs6651806	A	64.0%	76.7%	64.9%	77.8%
	C	36.0%	23.3%	35.1%	22.2%
Statistics		χ^2=4.201; df=2; **p=0.040**		χ^2=3.902; df=1; **p=0.048**	
MAO-B rs1799836 - rs6651806	AA	48.1%	58.7%	48.9%	59.7%
	GC	36.4%	23.9%	35.6%	22.2%
	GA	15.6%	17.4%	15.5%	18.1%
Statistics		χ^2=4.185; df=2; p=0.123		χ^2=4.247; df=2; p=0.120	

MAO-B—monoamine oxidase B.
